# Case Report: Genetic Double Strike: VEXAS and TET2-Positive Myelodysplastic Syndrome in a Patient With Long-Standing Refractory Autoinflammatory Disease

**DOI:** 10.3389/fimmu.2021.800149

**Published:** 2022-01-20

**Authors:** Fabian Lötscher, Luca Seitz, Helena Simeunovic, Adela-Cristina Sarbu, Naomi A. Porret, Laurence Feldmeyer, Luca Borradori, Nicolas Bonadies, Britta Maurer

**Affiliations:** ^1^ Department of Rheumatology and Immunology, Inselspital, Bern University Hospital, University of Bern, Bern, Switzerland; ^2^ Department of Hematology and Central Hematology Laboratory, Inselspital Bern, University of Bern, Bern, Switzerland; ^3^ Department of Dermatology, Inselspital, Bern University Hospital, University of Bern, Bern, Switzerland; ^4^ Department for BioMedical Research, University of Bern, Bern, Switzerland

**Keywords:** VEXAS syndrome, autoinflammation, TET2, vasculitis, MDS

## Abstract

Somatic genetic mutations involving the innate and inflammasome signaling are key drivers of the pathogenesis of myelodysplastic syndromes (MDS). Herein, we present a patient, who suffered from a long-standing refractory adult-onset autoinflammatory syndrome (AIS), previously interpreted as various distinct rheumatic disorders. Developing pancytopenia and particularly macrocytic anemia prompted the screening for a hematological malignancy, which led to the diagnosis of a *TET-2*-positive MDS. The impressive and continuously changing range of organ involvement, with remarkable refractoriness to anti-inflammatory treatment, exceeded the common autoinflammatory phenotype of MDS patients. This prompted us to suspect a recently discovered disease, characterized by somatic mutations of the *UBA1* gene: the VEXAS (Vacuoles, E1 enzyme, X-linked, Autoinflammatory, Somatic) syndrome, which was ultimately confirmed by genetic testing. Reevaluation of previous bone marrow biopsies showed the presence of characteristic vacuoles in myeloid- and erythroid progenitor cells. Our case illustrates that the triad of an unresponsive multisystemic autoinflammatory disease, hematological abnormalities and vacuoles in myeloid- and erythroid progenitors in the bone marrow biopsy should prompt screening for the VEXAS syndrome.

## Introduction

VEXAS (Vacuoles, E1 enzyme, X-linked, autoinflammatory, somatic) is a myeloid driven, adult-onset inflammatory syndrome associated with hematological neoplasms (1). The disease presents with a wide range of manifestations, including macrocytic anemia, fever and constitutional symptoms, neutrophilic dermatoses, cutaneous vasculitis and pulmonary infiltrates, making the diagnosis challenging. A characteristic finding in the bone marrow is vacuolization of the myeloid and erythroid progenitors. Before the first description of VEXAS, affected patients were diagnosed with a variety of conditions such as polyarthritis, giant cell arteritis, relapsing polychondritis (RP), polyarteritis nodosa, Sweet’s syndrome, myelodysplastic syndrome (MDS) and multiple myeloma. The syndrome is caused by somatic mutations in the Ubiquitinin-like modifier activating enzyme 1 gene (*UBA1*), which results in loss of function of this cytoplasmic enzyme, leading to systemic inflammation (1). The VEXAS syndrome is associated with MDS and rarely with multiple myeloma (1, 2). MDS itself can also be associated with refractory dysimmune and autoinflammatory manifestations, which may be exacerbated by concurrent VEXAS syndrome, however, the distinction between cause and consequence remains unclear (3–5). Conventional disease-modifying antirheumatic drugs and cytokine targeting therapies seem to have an inconstant and limited efficacy in VEXAS as well as in other patients with autoinflammatory manifestations related to MDS (6, 7).

We present a patient with long-standing corticosteroid dependent AIS, associated with a broad range of systemic manifestations, including seronegative polyarthritis and pancytopenia, ultimately diagnosed as VEXAS syndrome with MDS, originating from isolated *TET2* mutation with high allele burden.

## Case Description

**Figure 1:**
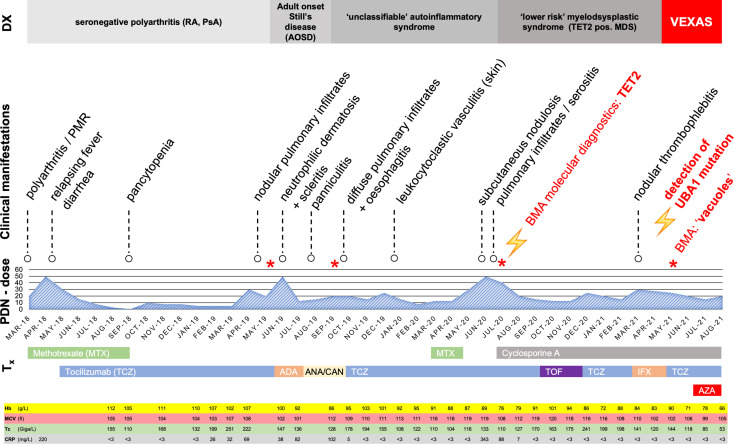
Overview with timeline of clinical manifestations and treatments March 2018 to August 2021. Tx, therapy; PDN-dose, prednisolone dose (in mg/day); DX, current working diagnosis; MTX, Methotrexate; TCZ, Tocilizumab; ADA, Adalimumab; ANA, Anakinra; AZA, Azacytidine; CAN, Canakinumab; TOF, Tofacitinib; IFX, Infliximab; BMA, bone marrow aspirate; PMR, polymyalgia rheumatica; RA, rheumatoid arthritis; PsA, psoriatic arthritis; Hb, Hemoglobin (normal range, 135-168 g/L); MCV, mean corpuscular volume (normal range, 80-89 fl); Tc, Thrombocytes (normal range,150-450 G/L); CRP, C-reactive protein (normal < 3 mg/L); *Bone marrow aspirate.

A 68-year-old male patient initially presented to his rheumatologist in early 2018 with polyarthritis of the ankles, wrists and fingers, polymyalgic symptoms and mild macrocytic anemia. Repeated detailed serologic evaluations for systemic autoimmune disorders and infections were unremarkable, including negative rheumatoid factor, ACPA, ANA and anti-PR3/MPO antibodies. Measures of CRP, hemoglobin, MCV and thrombocytes are shown in detail in **Figure 1**; minimum/maximum values in **Supplementary Table 1**. Imaging (MRI of the aorta and ultrasound of the temporal arteries) showed no evidence of vasculitis of the large vessels or solid malignancies. Seronegative rheumatoid arthritis (RA) was suspected and treatment with oral corticosteroids (CS) with up to 50 mg oral prednisone (PDN) daily and add-on methotrexate (MTX), 15 mg/week s.c. was initiated (**Figure 1**). The initial treatment response was favorable, especially to CS, but PDN dose reductions repeatedly led to arthritic flares and the development of new symptoms such as relapsing fever and diarrhea. After the repeated exclusion of infectious diseases, an additional therapy with tocilizumab (TCZ) was started. In late 2018, the patient developed pancytopenia and MTX was subsequently stopped as a potential contributing factor.

**Figure 2:**
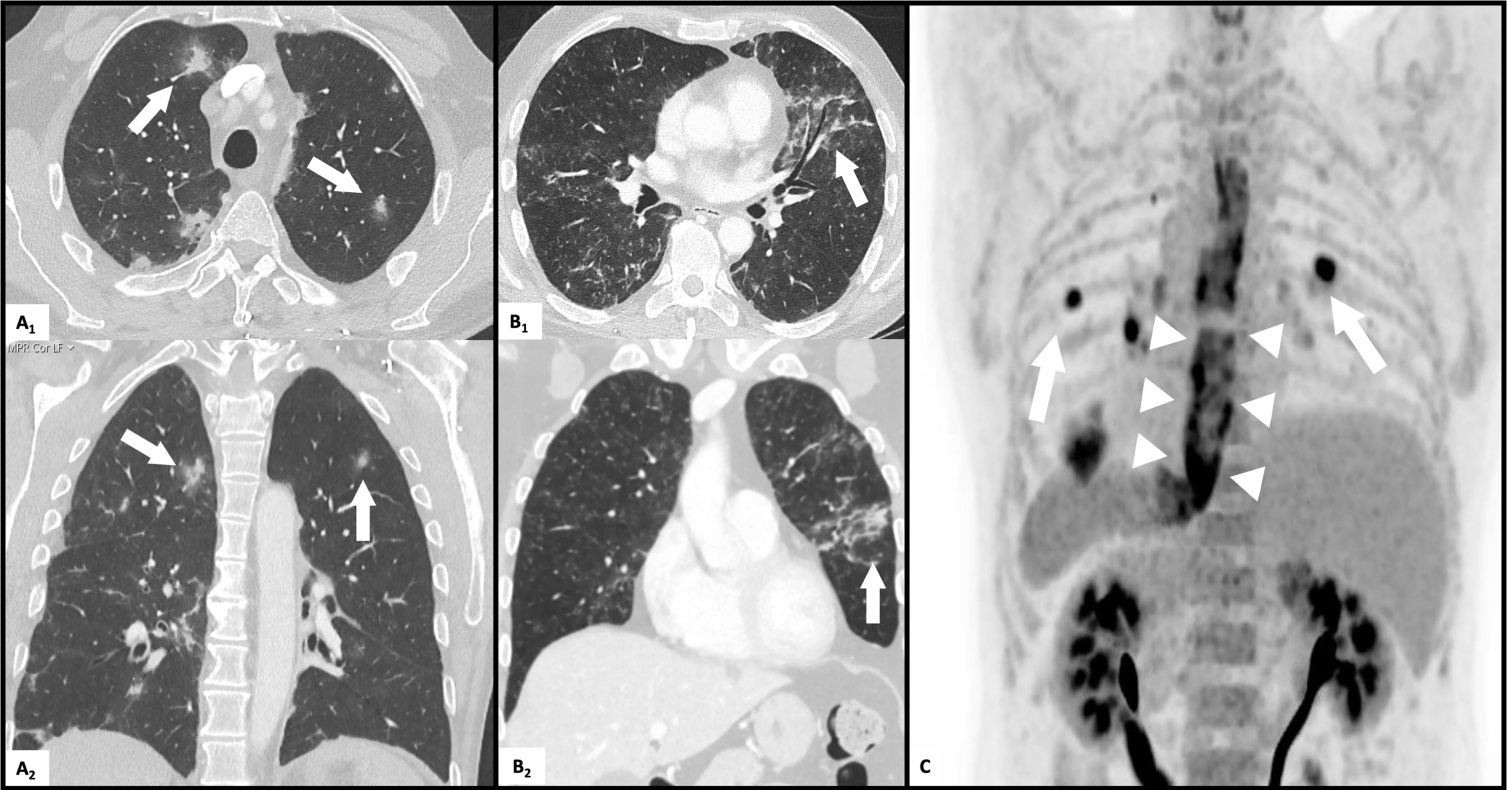
Pulmonary and esophageal affection on CT-scans. **(A)** multifocal nodular pulmonary infiltrates (arrows) on transverse **(A_1_)** and coronal **(A_2_)** high resolution CT scan. **(B)** diffuse pulmonary infiltrates (arrows) on transverse **(B_1_)** and coronal **(B_2_)** high resolution CT scan. **(C)** FDG-PET-CT scan: diffuse esophageal (arrowheads) tracer uptake and multiple pulmonary nodules (arrows).

**Figure 3:**
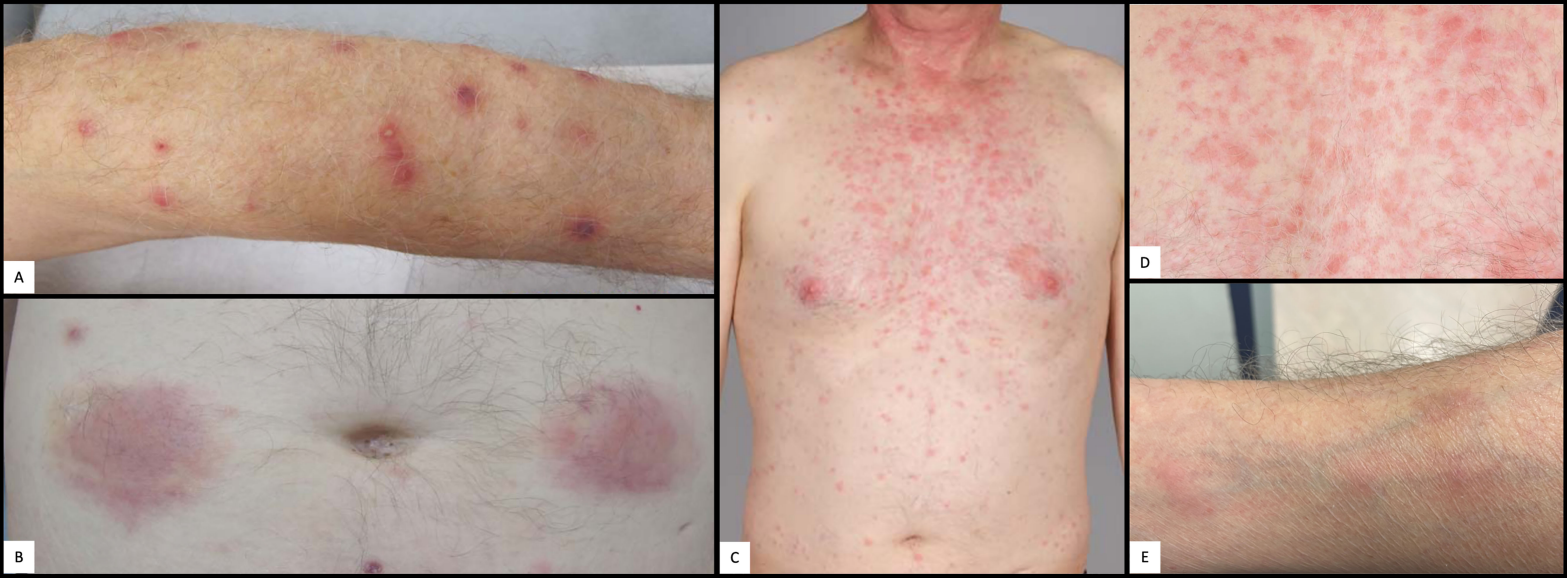
Cutaneous manifestations. **(A)** Pustulosis of the left arm. **(B)** Abdominal panniculitis after local application of anakinra. **(C)** Cervico-thoracic neutrophilic dermatosis. **(D)** Close up picture of cervico-thoracic neutrophilic dermatosis in **(C)**. **(E)** Nodular phlebitis of the right forearm.

**Figure 4:**
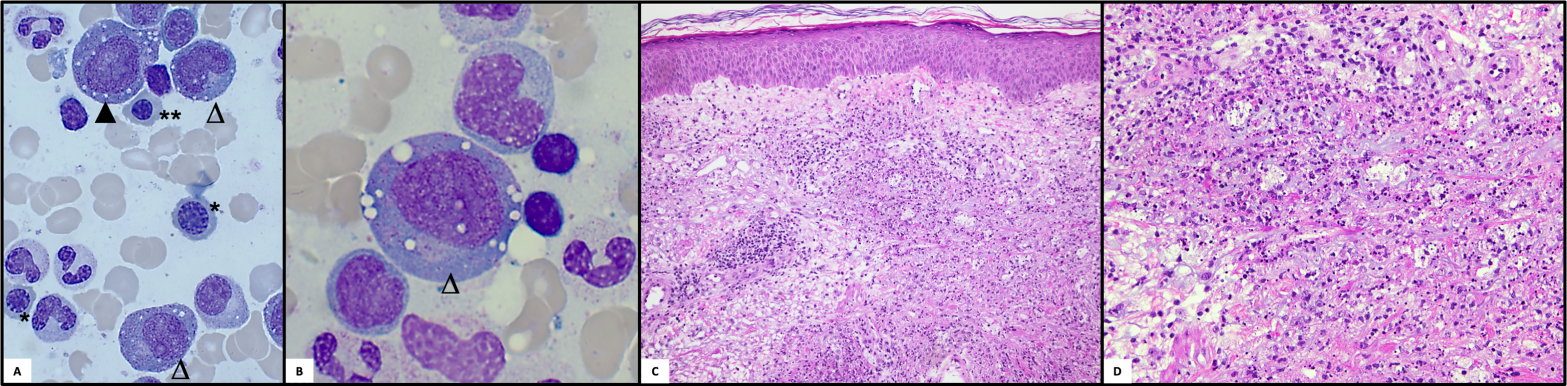
Histologic examinations. **(A)** Bone marrow cytomorphology: Pappenheim staining of a bone marrow aspirate is shown with typical vacuolizations in erythroid (▲) and myeloid progenitors (△). Moreover, megaloblastic (*) and macroblastic (**) changes can be seen in the normoblasts *(light microscope Zeiss, x40*). **(B)** Bone marrow cytomorphology: Higher magnification of vacuolized myeloid progenitor *(light microscope Zeiss, x100*). **(C, D)** Histology of the skin: Neutrophilic dermatosis with neutrophilic perivascular and interstitial infiltrate with leukocytoclasia and few eosinophils involving the dermis.

About one year later, the patient was hospitalized with pleurisy and nodular pulmonary infiltrates (**Figure 2A**). Infection was ruled out by bronchoscopy and bronchoalveolar lavage, and due to secondary therapy failure of TCZ, the TNF-inhibitor adalimumab (ADA) was initiated. Shortly thereafter, scleritis and a generalized neutrophilic dermatosis (dermatose neutrophilique en plaques, **Figures 3C, D** and **4C, D**) emerged, followed by sterile non-follicular pustular and papular eruptions on the upper chest and back, pubic area and extremities (**Figure 3A**) and after only two applications, ADA was stopped. Adult-onset Still’s disease (AOSD) was then postulated – although the Fautrel- and Yamaguchi-criteria were not fulfilled (8, 9). In the same period, CS responsive cytopenia persisted. An initial hematologic workup with bone marrow assessment showed multilineage reactive changes and normal cytogenetics, which was insufficient for a definitive diagnosis of MDS (**Figure 1**).

The CS dose was again escalated, and the patient was referred to our center in mid-2019, with elevated inflammatory markers despite intensified treatment (PDN 50mg daily) upon presentation. A yet unclassifiable autoinflammatory syndrome was suspected and the IL-1 antagonist anakinra (ANA), 100mg s.c. once daily, was started. The regular application of ANA led to a satisfactory relief of inflammatory symptoms and allowed considerable CS dose reduction, but provoked severe panniculitis at the injection sites (**Figure 3B**). Treatment was switched to canakinumab (one application of 150mg s.c., without subsequent local reaction of the skin), which led to an immediate deterioration with markedly elevated inflammatory markers, recurrence of pleurisy and new diffuse pulmonary infiltrates (**Figure 2B**). Again, infection was ruled out with bronchoscopy and bronchoalveolar lavage. An FDG-PET-CT scan revealed no signs of malignancy but showed significant esophageal tracer-uptake (**Figure 2C**), with no corresponding pathology detected on subsequent endoscopy of the upper gastrointestinal tract and MRI. With the diagnosis still being unclear and after a thorough review of the effectiveness of previous therapies, it was concluded that TCZ had shown the greatest therapeutic benefit. Thus, therapy was switched to TCZ again. Recurrent pulmonary and cutaneous symptoms and the inability to reduce the CS dose, led to a renewed attempt to add MTX to the therapeutic regimen. Due to worsening anemia with steady progression of the macrocytosis (maximal mean corpuscular volume: 120 fl), MTX was stopped again after a brief period. In addition, recurrent thrombocytopenia and leukopenia were observed, which persisted after stopping MTX and prompted a re-evaluation of the patient with regard to the presence of an underlying hematological disease. BMA was repeated twice (September 2019 and July 2020) and only the latest examination finally unmasked an MDS with multilineage dysplasia (MDS-MLD) by the detection of a *TET2* mutation [c.5681C>T, p.(Pro1894Leu] with a high variant allele frequency (VAF) of 47%. The unusually high VAF of the isolated *TET2* mutation in combination with the clinical course and morphological signs allowed the integrative diagnosis of MDS according to the minimal diagnostic criteria published by the international MDS working group (10). According to the Revised International Prognostic Scoring System (R-IPSS), the patient was classified in the lower-risk MDS category (11). Since MDS is known to be associated with dysimmune and autoinflammatory phenomena (4, 12, 13), the entire clinical presentation was initially assumed to be sufficiently explained by paraneoplastic phenomena – and cyclosporine A (CyA) was added to the installed TCZ. Due to refractory and symptomatic anemia, treatment with repetitive erythropoietin (EPO) injections was initiated but subsequently stopped due to ineffectiveness. The patient subsequently became red blood cell transfusion dependent.

Siglec-1 testing revealed a marked interferon signature, which led to the trial with a JAK-inhibitor: in November 2020 tofacitinib up to 20mg daily was initiated, with no clinical benefit during the 3 months applied. This was followed by a trial of infliximab (IFX) up to 5mg/kg bodyweight monthly for two months, with no relevant treatment response and even development of nodular phlebitis of several veins of both arms (**Figure 3E**). Meanwhile, the CS dependency persisted, and the patient eventually repeatedly developed symptoms such as phlebitis, alveolitis and recurrent scleritis even at relatively high PDN doses and resumed concomitant TCZ therapy. In October 2020, somatic mutations of *UBA1* and the associated VEXAS syndrome were described for the first time (1). The unusually severe and extremely refractory clinical course prompted us to screen for a concomitant somatic mutation in the *UBA1* gene. One of the previously described pathogenic mutations, pMet41Thr (c.122T>C), could be identified. At that point, a new BMA, performed for staging of the MDS, confirmed the presence of the typical vacuolisation of the myeloid and erythroid progenitor cells (**Figures 4A, B**). A retrospective analysis of all the patient’s BMA’s revealed that the previously deemed rather unspecific phenomenon of vacuolisation was already present before. Therapy was switched to a combination of TCZ and the hypomethylating agent azacytidine (AZA), since we did not expect AZA monotherapy to be sufficiently efficient for the inflammatory disease component. Both agents were applied in reduced dose to prevent an aggravation of cytopenia (TCZ: 4mg/kg bodyweight i.v. monthly; AZA: a total of 3 cycles: with 75mg/m^2^ body surface area s.c./d on five consecutive days in cycle 1 and 2, on day 1-5 and day 8 and 9 in cycle 3). This therapy was excellently tolerated, but the CS had to be increased again due to inflammatory activity (scleritis) and no effect on the cytopenias. The patient was finally evaluated for allogeneic hematopoietic stem cell transplantation (allo HSCT), as a potential curative therapeutic option.

## Discussion

The AIS in our patient had been progressing for years and was attributed to various diagnoses over time. The initially rather discreet but subsequently slowly worsening changes of the blood count - especially macrocytosis and intermittent thrombo-, leuco- and neutropenia – were only partially responsive to CS and concomitant immunosuppressive therapy. This led to the assumption of an underlying disease of the hematopoietic system, which could only be proven after serial bone marrow examinations including molecular genetic analyses. Since the 1990’s, MDS has been known to be associated with systemic dysimmune diseases (4, 5, 12, 13). Manifestations such as systemic vasculitides (e.g. giant cell arteritis, polyarteriitis nodosa or Behçet’s syndrome), relapsing polychondritis, neutrophilic dermatoses or connective tissue diseases (e.g. systemic lupus erythematodes, Sjögren’s syndrome) can precede or follow the diagnosis of MDS in more than one third of these patients (5, 7, 14).

It has been shown that certain loss of function mutations (for example affecting DNA methylation in *TET2* and *DNMT3A* mutations) result in upregulation of the pro-inflammatory cytokines interleukin 6 *(IL-6)* and interleukin 1 *(IL-1)* leading to systemic inflammation (15–17). In MDS, the prognostic significance of driver mutations is undisputed, but the prognostic impact of dysimmune/autoinflammatory manifestations continues to be debated (5, 14, 18, 19). While in a large cohort of patients with MDS, overall survival remained similar to patients without AIS, recent publications indicate a poorer prognosis for patients with dysimmune and autoinflammatory complications (14, 17, 18).

In our patient, we found an unusually high allele burden of an isolated *TET2* mutation, which raises the question of the possibility of an underlying germline variant. This specific mutation has been described, even though it is rare, in the COSMIC database (Catalogue of Somatic Mutations in Cancer), within a somatic context. It is not present in the gnomAD database (Genome Aggregation Database). In a potential germline context, it would be classified as a variant of unknown significance. As a somatic variant makes sense for MDS, the germline context was not further investigated. However, the severity and treatment refractoriness of the autoinflammation seemed not to be explained solely by the *TET2* mutation, which prompted us to search for the recently described VEXAS syndrome. The hallmark of the VEXAS syndrome is a treatment refractory, myeloid driven inflammatory syndrome with the appearance of complex rheumatic diseases as well as profound hematologic abnormalities (1). Clonal hematologic disorders are reported in up to 50% of VEXAS patients (1–3, 20). An overview of the current literature about VEXAS syndrome and concomitant clonal hematological disorders is presented in **Supplementary Table 2**. Important aspects are briefly summarized below. So far, 106 patients with VEXAS and MDS could be identified. Plasma cell dyscrasias, such as MGUS (14 subjects) and multiple myeloma (3 patients), and myelofibrosis (1 subject) were rarely observed. Characteristic somatic mutations were present in 1/3 of the patients (38/106), most frequently DNMT3A (22%, 23/106) and TET2 (11%, 12/106). Additional somatic mutations (transcription factor mutations TP53 or RUNX1, as well of other genes including CBL, KRAS, NRAS, ZRSR2 and EP300) were mostly present in combination with TET2 or DNMT3A mutations. Interestingly, retrospective studies including subjects with chronic myelomonocytic leukemia (CMML), who frequently have TET2 mutations, did not reveal any cases with concomitant UBA1 mutation (21, 22). A retrospective French study revealed, that patients with VEXAS and MDS were more likely to have fever, pulmonary infiltrates and arthritis, had lower platelet counts, received more CS, but had the same survival rate as VEXAS patients without MDS. Furthermore, they identified 3 different clusters: (1) mild-to-moderate disease, with reduced incidence of fever, chondritis and thromboembolism, (2) MDS related phenotype, with relapsing chondritis, fever and venous thromboembolism, and (3) an ‘inflammatory’ profile, with older patients, more frequent weight loss, cutaneous vasculitis and higher median CRP levels. (2) Patients in cluster 2 more frequently received azacytidine and had the highest mortality rates. UBA1 p.Met41Leu mutations were associated with a less inflammatory and mild-to moderate phenotype. If these aspects are confirmed in larger number of VEXAS patients, this may allow the classification into high and low risk VEXAS and could influence future treatment decisions.

Our case illustrates that vacuolisation, which is a rather unspecific finding observed in MDS, acute myeloid leukemia as well as in zinc deficiency and others, must be searched explicitly (23, 24). The retrospective analysis of the previous BMA revealed the presence of significant vacuolisation two years prior to the VEXAS diagnosis (**Figures 4A, B**), but initially it was not reported until the very last BMA since previously deemed unspecific and irrelevant. Vacuolisation of myeloid and erythroid precursor cells could indicate VEXAS if a certain proportion (>10%) of neutrophil precursors in the BMA are affected and should prompt genetic testing for *UBA1* in the appropriate clinical setting of uncharacterized and systemic autoinflammatory manifestations (23).

AIS in patients with MDS pose a therapeutic challenge. AIS generally seem to be CS responsive, but with a considerable CS dependency and morbidity. Most patients are in need for CS-sparing immunosuppressive treatments, usually requiring the use of second- and even third-line agents with various modes of action. This is well illustrated by our case. High doses of CS were necessary to control the disease and multiple conventional, biological and small molecule immunosuppressive agents were used in mono- or combination therapy (**Figure 1**). While TNF-inhibitors and MTX or CyA did not influence disease activity, symptoms always responded well to doses of prednisone > 20-25 mg/day. At least temporarily, a CS-sparing effect of TCZ and ANA could be observed, but the latter very quickly led to pronounced local panniculitis, which was also observed in the cohort of Beck and colleagues (1). In 2017, Mekinian et al. were able to illustrate in a small retrospective study that multi-line therapies are usually necessary and that most biologic therapies applied do not show a long-lasting effect in patients with SIAD and MDS. Azacytidine, a hypomethylating agent, frequently used in the treatment of higher-risk MDS, was the most promising therapeutic agent in this study and was used for two cycles for our patient as part of a bridging procedure to allo HSCT but without relevant improvement of the inflammatory manifestations (7). Regarding the VEXAS syndrome, all major cohorts published to date indicate that the inflammatory disease manifestations are CS-sensitive but poorly responsive to other anti-inflammatory therapeutic strategies. In particular, changes in the blood count appear to be rather resistant to therapy, with progression over time. The resistance to multiple targeted immunosuppressive therapies and the manifold clinical manifestations, may be due to the simultaneous upregulation of various cytokines described in the original cohort (1). A French center described a promising response to JAK-inhibitor therapy: 2 patients were treated with the JAK1/2 inhibitor ruxolitinib, and 1 patient with the JAK1/3 inhibitor tofacitinib. The IFN-signature of our patient as indicated by Siglec-1-positivity as well as the option to target multiple cytokines prompted us to follow this approach. In our patient, however, tofacitinib did not prove beneficial. This might be due to the concomitant MDS with *TET2* mutation, the late stage of the disease or the choice of the JAK-inhibitor (6, 25). Allo HSCT aims to eradicate the clonal cells responsible for the disease and thus has the potential to cure patients with treatment refractory VEXAS syndrome as well as associated MDS. A recently published retrospective analysis of 6 patients with VEXAS and allo HSCT is promising, with 3 patients in durable remission, 2 alive in short-term follow up, and 1 death HSCT-associated infection (26). However, prospective data are lacking for the individual assessment of this resource intensive therapy with a risk of additional long-lasting immunological complications (GvHD, infections). A first phase II study has been launched (ClinicalTrials.gov Identifier: NCT05027945).

## Conclusion

The recent description of the VEXAS syndrome sheds light on patients with longstanding refractory AIS, frequently accompanied by MDS and opens the door to a better understanding of the interplay between the immune system and clonal hematopoietic cells. This case demonstrates that in elderly patients with AIS and concomitant hematological manifestations, an extensive and, if necessary, repetitive diagnostic work-up, including molecular testing for myeloid driver mutations and *UBA1*, is indicated and justified. Furthermore, it illustrates the broad clinical spectrum of this syndrome, as well as its therapeutic challenges, which demands an interdisciplinary management and treatment strategy. Specialists in the fields of rheumatology, immunology, hematology, oncology, dermatology and general internal medicine should be aware of this syndrome and patients should be included in prospective clinical trials to improve the diagnostic approach, risk assessment and enable effective treatment strategies in the near future.

## Data Availability Statement

The original contributions presented in the study are included in the article/**Supplementary Material**. Further inquiries can be directed to the corresponding author.

## Ethics Statement

Ethical review and approval was not required for the study on human participants in accordance with the local legislation and institutional requirements. Written informed consent of the patient is available for the publication of this case-report and the inclusion in the Swiss MDS Registry (CEC: 2016-01917).

## Author Contributions

FL, LS, LB, NB, and BM were involved with the conception of the work. FL, LS, HS, AS, NP, and LF contributed to the acquisition of data. FL, LS, NP, LF, LB, NB, and BM contributed to the analysis and interpretation of the data. FL and LS drafted the manuscript. FL, LS, NP, NB, and BM participated in the revision of the manuscript. All authors approved of the submitted version.

## Conflict of Interest

The authors declare no conflict of interest for the interpretation of the survey.

Potentially perceived conflicts of interests according to the definitions and terms of International Committee of Medical Journal Editors are:

NB: Alexion: research funding to institution; Amgen: financial support for travel; Astellas: research funding to institution; Celgene/BMS: financial support for travel, research funding to institution; consultancy honoraria; Janssen: financial support for travel; Novartis: financial support for travel, research funding to institution, consultancy honoraria; Roche: financial support for travel, research funding to institution; Sandoz: research funding to institution; Servier: research funding to institution; Takeda: research funding to institution.

BM has consultancies with Novartis, Boehringer Ingelheim, Janssen-Cilag, had grant/research support from AbbVie, Protagen, Novartis Biomedical Research, received speaker fees from Boehringer-Ingelheim as well as congress support from Medtalk, Pfizer, Roche, Actelion, Mepha, and MSD. In addition, BM has a patent mir-29 for the treatment of systemic sclerosis issued (US8247389, EP2331143).

## Publisher’s Note

All claims expressed in this article are solely those of the authors and do not necessarily represent those of their affiliated organizations, or those of the publisher, the editors and the reviewers. Any product that may be evaluated in this article, or claim that may be made by its manufacturer, is not guaranteed or endorsed by the publisher.

## Abbreviations

ADA, Adalimumab; AIS Adult-onset inflammatory syndrome; allo HSCT, Allogeneic hematopoietic stem cell transplantation; AOSD, Adult-onset Still’s disease; AZA, Azacytidine; CS, Corticosteroids; CyA, Cyclosporine A; DMARD, Disease modifying antirheumatic drug; IFX, Infliximab; IL-1, Interleukin 1; IL-6, Interleukin 6; IPSS, International prognostic scoring system; MGUS, Monoclonal gammopathy of undetermined significance; MDS, Myelodysplastic syndrome; MLD, Multilineage dysplasia; MTX, Methotrexate; PDN, Prednisolone; RA, Rheumatoid arthritis; RP, Relapsing polychondritis; SIGLEC, 1Sialic acid binding Ig-like lectin 1; TNF, Tumor necrosis factor; TCZ, Tocilizumab; UBA1, Ubiquitin-like modifier activating enzyme 1; VEXAS, Vacuoles, E1 enzyme, X-linked, Autoinflammatory, Somatic; VAF, Variant allele frequency.
