# Supplementary material for: Case Report: Genetic Double Strike: VEXAS and TET2-Positive Myelodysplastic Syndrome in a Patient With Long-Standing Refractory Autoinflammatory Disease
Source: Front Immunol. 2022 Jan 20;12:800149. doi: 10.3389/fimmu.2021.800149 (PMC8811255; doi:10.3389/fimmu.2021.800149)
Supplement: Supplementary file 1 [file DataSheet_1.docx]

**Supplementary Data:**

**Supplementary Methods**

**NGS MDS Panel:**

Molecular analysis for MDS was performed with the Oncomine™ Myeloid Research Assay using the Ion Torrent S5 instrument. It covers 40 relevant myeloid genes, and with a sensitivity of 5% VAF (variant allele frequency), and which is not error corrected. The following genes were analyzed:

ABL1 (Exone 4-9), ASXL1 (Exone 11-12), BCOR, BRAF (Exone 11, 15), CALR, CBL (Exone 8-9), CEBPA, CSF3R (Hotspots in Exone 14, 17, 18), DNMT3A (Exone 11-23), ETV6, EZH2, FLT3 (Exone 8, 11, 14-16, 21), GATA2 (Hotspots in Exone 4, 5), HRAS (Exone 2, 3), IDH1 (Hotspot in Exon 4), IDH2 (Hotspot in Exon 4), IKZF1, JAK2 (Exone 12-15), KIT (Hotspots in Exone 8. 9, 10, 11, 13, 17), KRAS (Hotspots in Exone 2, 3, 4), MPL (Hotspots in Exone 3, 4, 10, 12), MYD88 (Exone 3, 5), NF1, NPM1 (Exon 11), NRAS (Hotspots in Exone 2, 3, 4), PHF6, PRPF8, PTPN11 (Hotspots in Exone 3, 12, 13), RB1, RUNX1, SETBP1 (Hotspot in Exon 4), SF3B1 (Hotspots in Exone 14, 15, 16, Exone 17-21), SH2B3, SRSF2 (Exon 1), STAG2, TET2, TP53, U2AF1 (Hotspots in Exone 2, 6), WT1 (Exone 6, 8), ZRSR2.

**Supplementary Tables:**

**Supplementary Table 1: Overview of laboratory parameters and autoimmune serologies**

| **Parameter (Unit)** | **Normal range** | **Patients’ max.** | **Patients’ min.** |
| --- | --- | --- | --- |
| CRP (mg/L) | < 3 | 343 | <3 |
| Ferritin (ug/L) | 20 - 250 | 3008 | 355 |
| Hemoglobin (g/L) | 135 - 168 | 112 | 66 |
| MCV (fl) | 80 - 98 | 120 | 99 |
| Thrombocytes (Giga/L) | 150 - 450 | 251 | 85 |
| Leukocytes (Giga/L) | 3.00 - 10.50 | 10.8 | 1.95 |
| Neutrophils (Giga/L) | 1.60 - 7.40 | 11.96 | 0.71 |
| Rheumatoid Factor IU/ml | < 3.50 | 0.5 |  |
| ACPA | neg. | neg. |  |
| ANA | < 1:80 | < 1:80 |  |
| Anti-PR3 Abs. (IU) | < 5 | < 0.5 |  |
| Anti-MPO Abs. (IU) | < 6 | < 1 |  |

**Supplementary Table 1**: Laboratory parameters and autoimmune serologies. Only normal range, minimal and maximal values are listed**.**

**Supplementary Table 2: Formal review of the literature, describing VEXAS patients with concomitant clonal hematologic disorders**

| **Author (Reference)** | **MDS** | **MDS-associated somatic mutations** | | | **Other diseases** | **Comments** |
| --- | --- | --- | --- | --- | --- | --- |
|  |  | *TET 2* | *DNMT3A* | Other |  |  |
| Oganesyan et al. 03/2021 (23) | 1 |  | 1 | 1 *EP300* |  | *EP300* concomitant with *DNMT3A* |
| Rieu et al. 03/2021 (22) | 1 |  |  |  |  | MDS, no additional somatic mutations |
| Tsuchida et al. 03/2021 (20) | 6 |  |  |  |  | RP-VEXAS and MDS in 6 subjects, no additional somatic mutations |
| Huang et al. 03/2021 (10) | 1 |  |  |  |  | MDS, no additional somatic mutations |
| Sakuma et al. 04/2021 (21) | 1 |  |  |  |  | MDS, no additional somatic mutations |
| Staels et al. 04/2021 (9) | 1 |  | 1 |  |  |  |
| Van der Made et al. 05/2021 (16) | 4 |  | 1 |  |  |  |
| Lytle et al. 06/2021(18) | 1 |  |  |  |  | MDS with *CCND1-IGH* |
| Templé et al. 07/ 2021 (19) | 2 |  |  | 1 ***PPM1D*** |  | 2 Subjects with MDS; Atypical UBA1 splice site mutation; NO vacuoles |
| Obiorah et al. 08/2021 (6) | 6 |  | 2 | ***1 GNA11+CSF1R***  *(1 EZH2)* | 2 MM  2 MBL  2 MGUS | 2 MGUS (1 with concomitant MM)  MBL: monoclonal B-Cell lymphocytosis  EZH2 in subject without MDS diagnosis |
| L.Zhao et. al. 08/2021 (3) | 4 | 1 | 1 | *1 TP53* |  | *TP53* concomitant with *DNMT3A* |
| Dupuy et al. 09/2021 (8) |  |  |  |  |  | *No UBA1 mutations in a cohort of 108 CMML Patients* |
| Shaukat et al. 09/2021 (2) | 1 |  | 1 |  |  | Review of the literature: 31% of VEXAS patients with MDS, 7% with MGUS, 1% with MM |
| Li et al. 10/2021 (13) | 0 |  |  | 1 ***PRPF40B*** |  | 2 subjects without MDS; germline origin suggested (VAF 48%) |
| Muratore et al. 10/2021 (14) | 5 |  |  |  |  | 5 subjects with MDS and no cytogenetic changes; all with vasculitis |
| Raaijmakers et al. 10/2021 (1) | 3 | 1 | 2 |  |  | 3 additional patients in cohort, but no specific details provided |
| Georgin-Lavialle et al. 10/2021 (4) | 52 | 6 | 11 | 1 unclear | 12 MGUS (all with MDS) | 52 MDS (out of 116 VEXAS Patients)  Identification of 3 clusters as well as phenotype association for *UBA1 p.MET41Leu* (less inflammatory, better prognosis) |
| Pamies et al. 10/2021 (12) | 1 |  |  |  |  | RP-VEXAS with MDS + IgA Vasculitis |
| Diarra et al. 10/2021(5) | 5 | 2 | 1 | *1(CBL, KRAS*  *NRAS, ZRSR2)*  *1 RUNX1* | 1 Myelofibrosis | 6 HSCT Patients, retrospective analysis  all with additional mutations (*TET2, CBL, KRAS, NRAS, ZRSR2*) in 1 Patient with *TET2+* MDS  RUNX1 in Patient with *DNMT3A* mutation |
| Roy L. Kao et al. 11/2021 (11) | 0 |  | 1 |  |  | no MDS |
| Gurnari et al. 2021 (7) | 2 |  | 1 |  |  | Retrospective analysis of subjects with vacuolization on bone marrow examination |
| Koster et al. 2021 (15) | 1 | 1 |  |  | 1 MM |  |
| Grey et al. 2021 (17) | 1 |  |  |  |  | MDS, no additional somatic mutations |
| Bourbon et al. 2021 (24) | 6 |  |  |  |  | 6 MDS, no additional somatic mutations |
| Lötscher et al. 12/2021 | 1 | 1 |  |  |  |  |
| **24 Publications**  **including presented case-report** | **106** | **12** | **23** | **Combined w. TET2 or DNMT3A: 4**  **Isolated: 3** | **14 MGUS (13 with MDS, 1 with MM)**  **3 MM**  **1 Myelofibrosis** |  |
|  |  |  |  |  |  |  |

**Abbreviations:** VAF: variant allele frequency; MDS: myelodysplastic syndrome; MGUS: monoclonal gammopathy of undetermined significance; MBL: monoclonal B-Cell lymphocytosis; CMML: chronic myelomonocytic leukemia; MM: multiple myeloma; RP-VEXAS: relapsing polychondritis VEXAS. The literature search was performed via pubmed (search term: VEXAS syndrome) on 10 December 2021: 59 manuscripts were identified and each manuscript was screened for patients with clonal heamatologic diseases.

**References for supplementary Table 2:**

1. Raaijmakers MHGP, Hermans M, Aalbers A, Rijken M, Dalm VASH, Daele P Van, et al. Azacytidine Treatment for VEXAS Syndrome. Hemasphere. 2021;Nov 17(5(12)).

2. Shaukat F, Hart M, Burns T, Bansal P. UBA1 and DNMT3A mutations in VEXAS syndrome. A case report and literature review. Mod Rheumatol Case Reports. 2021;(July):1–6.

3. Zhao LP, Schell B, Sébert M, Kim R, Lemaire P, Boy M, et al. Prevalence of UBA1 mutations in MDS/CMML patients with systemic inflammatory and auto-immune disease. Leukemia. 2021;35(9):2731–3.

4. Georgin‐Lavialle S, Terrier B, Guedon AF, Heiblig M, Comont T, Lazaro E, et al. Further characterization of clinical and laboratory features occurring in VEXAS syndrome in a large‐scale analysis of multicenter case‐series of 116 French patients. Br J Dermatol. 2021;1–11.

5. Diarra A, Regional CH, Duployez N, Lille CHU, Fournier E, Preudhomme C, et al. Successful allogeneic hematopoietic stem cell transplantation in patients with VEXAS syndrome : a two center experience. Blood Adv. 2021;Oct 29.

6. Obiorah IE, Patel BA, Groarke EM, Wang W, Trick M, Ombrello AK, et al. Benign and malignant hematologic manifestations in patients with VEXAS syndrome due to somatic mutations in UBA1. Blood. 2021;5(16):3203–15.

7. Gurnari C, Pagliuca S, Durkin L, Terkawi L, Awada H, Kongkiatkamon S, et al. Vacuolization of hematopoietic precursors: an enigma with multiple etiologies. Blood [Internet]. 2021;137(26):3685–9. Available from: http://dx.doi.org/10.1182/blood.2021010811

8. Dupuy H, Dussiau C, Bidet A, Sauvezie M, De-Grande AC, Decombe J, et al. Looking for somatic mutations in UBA1 in patients with chronic myelomonocytic leukemia associated with systemic inflammation and autoimmune diseases. Leuk Lymphoma [Internet]. 2021;1–3. Available from: https://doi.org/10.1080/10428194.2021.1973674

9. Staels F, Betrains A, Woei-A-Jin S, Boeckx N, Beckers M, Bervoets A, et al. Case Report: VEXAS Syndrome: From Mild Symptoms to Life-Threatening Macrophage Activation Syndrome. Front Immunol. 2021;12(April):1–5.

10. Huang H, Zhang W, Cai W, Liu J, Wang H, Qin T, et al. VEXAS syndrome in myelodysplastic syndrome with autoimmune disorder. Exp Hematol Oncol [Internet]. 2021;10(1):1–5. Available from: https://doi.org/10.1186/s40164-021-00217-2

11. Kao RL, Jacobsen AA, Billington CJ, Yohe SL, Beckman AK, Vercellotti GM, et al. A case of VEXAS syndrome associated with EBV-associated hemophagocytic lymphohistiocytosis. Blood Cells, Mol Dis [Internet]. 2021;93(November 2021):102636. Available from: https://doi.org/10.1016/j.bcmd.2021.102636

12. Pàmies A, Ferràs P, Bellaubí-Pallarés N, Giménez T, Raventós A CR. VEXAS syndrome: relapsing polychondritis and myelodysplastic syndrome with associated im- munoglobulin A vasculitis. Rheumatol. 2021;Oct 20.

13. Li P, Venkatachalam S, Cordona DO, Wilson L, Kovacsovics T, Karen A, et al. A clinical , histopathological , and molecular study of two cases of VEXAS syndrome without a definitive myeloid neoplasm A clinical , histopathological , and molecular study of two cases of VEXAS syndrome without a definitive myeloid neoplasm Division of. Blood Adv. 2021;Oct 14.

14. Muratore F, Marvisi C, Castrignanò P, Nicoli D, Farnetti E, Bonanno O, Longo R, Zaldini P, Galli E, Balanda N, Beck DB, Grayson PC, Pipitone N, Boiardi L SC. VEXAS syndrome : a case series from a single-center cohort of Italian patients with vasculitis. Arthritis Rheumatol. 2021;Oct 5.

15. Koster MJ, Kourelis T, Reichard KK, Kermani TA, Beck DB, Cardona DO, et al. Clinical Heterogeneity of the VEXAS Syndrome. Mayo Clin Proc [Internet]. 2021;1–7. Available from: https://doi.org/10.1016/j.mayocp.2021.06.006

16. van der Made CI, Potjewijd J, Hoogstins A, Willems HPJ, Kwakernaak AJ, de Sevaux RGL, et al. Adult-onset autoinflammation caused by somatic mutations in UBA1: A Dutch case series of patients with VEXAS. J Allergy Clin Immunol [Internet]. 2021; Available from: https://doi.org/10.1016/j.jaci.2021.05.014

17. Grey A, Cheong PL, Lee FJ, Abadir E, Favaloro J, Yang S, et al. A Case of VEXAS Syndrome Complicated by Hemophagocytic Lymphohistiocytosis. J Clin Immunol [Internet]. 2021;41(7):1648–51. Available from: https://doi.org/10.1007/s10875-021-01070-y

18. Lytle A, Bagg A. VEXAS: a vivid new syndrome associated with vacuoles in various hematopoietic cells. Blood [Internet]. 2021 [cited 2021 Dec 14];137(26):3690. Available from: http://www.ncbi.nlm.nih.gov/pubmed/34196684

19. Templé M, Duroyon E, Croizier C, Rossignol J, Huet T, Friedrich C, et al. Atypical splice site mutations causing VEXAS syndrome. Rheumatology. 2021;Dec 1;60(12):e435–7.

20. Tsuchida N, Kunishita Y, Uchiyama Y, Kirino Y, Enaka M, Yamaguchi Y, et al. Pathogenic UBA1 variants associated with VEXAS syndrome in Japanese patients with relapsing polychondritis. Ann Rheum Dis. 2021;1–5.

21. Sakuma M, Tanimura A, Yasui S, Ishiguro K, Kobayashi T, Ohshiro Y, et al. A Case of polychondritis-onset refractory organizing pneumonia with cytopaenia diagnosed as VEXAS syndrome: The disease course of 7 years. Rheumatol (United Kingdom). 2021;60(10):E356–9.

22. Rieu JB, El Kassir A, Largeaud L, Dion J, Comont T, Mansat-De Mas V. Characteristic vacuolisation of granulocytic and erythroid precursors associated with VEXAS syndrome. Br J Haematol. 2021;194(1):8.

23. Oganesyan A, Jachiet V, Chasset F, Hirsch P, Hage-Sleiman M, Fabiani B, et al. VEXAS syndrome: still expanding the clinical phenotype. Rheumatology (Oxford). 2021;60(9):e321–3.

24. Bourbon E, Heiblig M, Gerfaud Valentin M, Barba T, Durel CA, Lega JC, Barraco F, Sève P, Jamilloux Y SP. Therapeutic options in VEXAS syndrome: insights from a retrospective series. Blood. 2021;137(26):3682–4.
